# Chemogenomic and transcriptome analysis identifies mode of action of the chemosensitizing agent CTBT (7-chlorotetrazolo[5,1-c]benzo[1,2,4]triazine)

**DOI:** 10.1186/1471-2164-11-153

**Published:** 2010-03-04

**Authors:** Monika Batova, Vlasta Klobucnikova, Zuzana Oblasova, Juraj Gregan, Pavol Zahradnik, Ivan Hapala, Julius Subik, Christoph Schüller

**Affiliations:** 1 Comenius University in Bratislava, Department of Microbiology and Virology, 842 15 Bratislava, Slovak Republic; 2 Slovak Academy of Sciences, Institute of Animal Genetics and Biochemistry, 90028 Ivanka pri Dunaji, Slovak Republic; 3 University of Vienna, Max F Perutz Laboratories, Department of Chromosome Biology, A-1030 Vienna, Austria; 4 Comenius University in Bratislava, Department of Organic Chemistry, 512 15 Bratislava, Slovak Republic; 5 University of Vienna, Max F Perutz Laboratories, Department of Biochemistry and Cell Biology, A-1030 Vienna, Austria

## Abstract

**Background:**

CTBT (7-chlorotetrazolo [5,1-c]benzo[1,2,4]triazine) increases efficacy of commonly used antifungal agents by an unknown mechanism. It increases the susceptibility of *Saccharomyces cerevisiae, Candida albicans *and *Candida glabrata *cells to cycloheximide, 5-fluorocytosine and azole antimycotic drugs. Here we elucidate CTBT mode of action with a combination of systematic genetic and transcriptome analysis.

**Results:**

To identify the cellular processes affected by CTBT, we screened the systematic haploid deletion mutant collection for CTBT sensitive mutants. We identified 169 hypersensitive deletion mutants. The deleted genes encode proteins mainly involved in mitochondrial functions, DNA repair, transcription and chromatin remodeling, and oxidative stress response. We found that the susceptibility of yeast cells to CTBT depends on molecular oxygen. Transcriptome analysis of the immediate early response to CTBT revealed rapid induction of oxidant and stress response defense genes. Many of these genes depend on the transcription factors Yap1 and Cin5. Yap1 accumulates rapidly in the nucleus in CTBT treated cells suggesting acute oxidative stress. Moreover, molecular calculations supported a superoxide generating activity of CTBT. Superoxide production *in vivo *by CTBT was found associated to mitochondria as indicated by oxidation of MitoSOX Red.

**Conclusion:**

We conclude that CTBT causes intracellular superoxide production and oxidative stress in fungal cells and is thus enhancing antimycotic drug effects by a secondary stress.

## Background

Fugal pathogens pose a serious threat to immunocompromised persons. Despite many antifungal agents interfering with metabolism and growth of fungal cells a limited number of compounds are being used for treatment of mycotic diseases caused by human pathogenic fungal species. Over the past two decades, the number of invasive fungal infections has increased in the clinical setting. *Candida sp*. is the fourth most common pathogen identified, and other pathogens such as *Cryptococcus sp., Aspergillus sp*., and *Fusarium sp*., have a high morbidity and mortality. In addition, the incidence of mycoses caused by opportunistic fungi is rising [1]. CTBT, 7-chlorotetrazolo [5,1-c]benzo[1,2,4]triazine, has antifungal activity and enhances the efficacy of other antifungals with different targets such as cycloheximide, fluconazole or 5-fluorocytosine [2]. The molecular mechanism of CTBT action has not yet been resolved.

Currently used antifungals belong to three major classes of agents: azoles, polyenes, and echinocandines [3]. These compounds target ergosterol biosynthesis, membrane functions and cell wall biosynthesis. Additionally, other currently applied compounds are the pyrimidine, 5-fluorocytosine (5-FC), which acts by inhibiting RNA and DNA synthesis [4] and ciclopiroxolamine which seems to induce oxidative stress and iron deprivation [5]. The intrinsic resistance of human fungal pathogens to these substances is different. Echinocandins are effective in *Candida *prevention and offer a greater spectrum of activity across the various *Candida *species, including also *C. krusei *and *C. glabrata*, which are not reliably covered by azoles e.g. fluconazole [6].

Fungal drug resistance mechanisms involve decreased drug uptake, increased drug export, overexpression or structural modification of the drug target protein [7,8]. Reversal of antifungal drug effectiveness in yeast cells mediated by efflux has been reported for a variety of substances targeting different molecular processes. These are for example the immunosuppressive agents FK506 and cyclosporine [9-13]. To overcome drug resistance of human fungal pathogens, new antifungals with novel cellular targets [14] and multidrug resistance reversal agents rendering drug resistant strains sensitive to commercially used antifungals are being developed [15,16] but have not surfaced as yet. Studies evaluating combinations of antifungals have shown synergistic and additive activity. However, caution is required, because some antifungal combinations have demonstrated antagonistic activity. Controlled clinical trials are still necessary to explore the various efficacious antifungal combinations [17]. Since the common antifungals are mainly targeting membrane and cell wall components, efficient combination therapy might be reached by involving substances with an alternative mode of action.

The site and mode of CTBT action have not yet been resolved. CTBT displayed a weak antifungal activity which was unaffected by deletion of the *PDR1 *and *PDR3 *genes encoding the main transcription activators involved in the control of multidrug resistance in *Saccharomyces cerevisiae *[18,19]. Yeast cells grown in its presence had altered sterol composition and were more sensitive to this compound in the *yap1Δ *genetic background [2]. Here we report insights gained for the mode of CTBT action by systematic identification of yeast genes required for resistance to CTBT in combination with transcriptome analysis. We will show that CTBT causes an unexpected dramatic response to oxidative stress including damage to mitochondria and genomic DNA. These results provide a model for CTBT action and indicate that its synergic effect with commonly used antifungal drugs is due to the combination of oxidative and other stresses.

## Results

### CTBT action depends on molecular oxygen and is connected by mitochondrial functions

**Figure 1:**
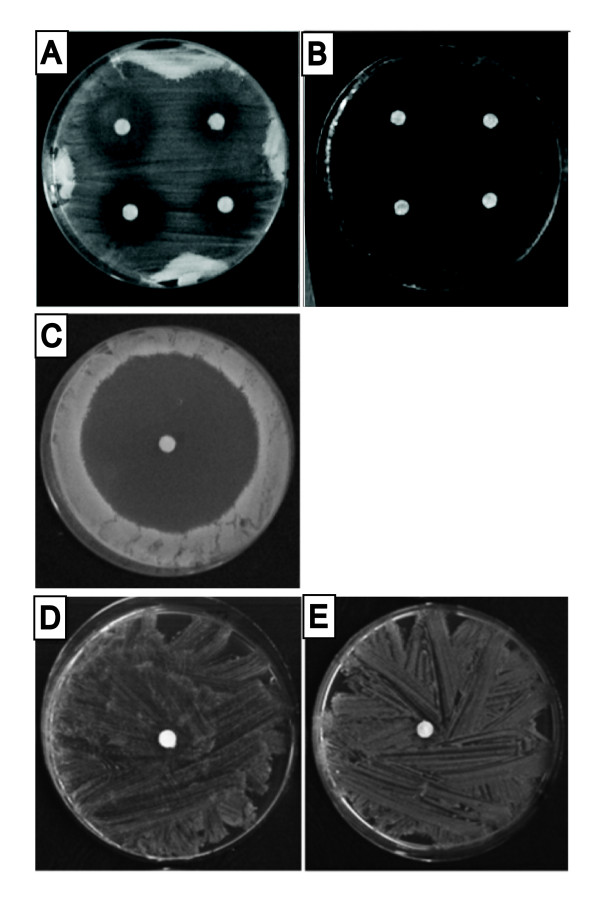
**CTBT induced growth inhibition zones on complex media. *S. cerevisiae *BY4741 wild type on YPD**. (A), BY4741 *rho*^- ^mutant on YPD (B), BY4741 on YPGE (C), BY4741 (D) and BY4742 (E) grown anaerobically on YPD supplemented with ergosterol plus Tween 80. Amounts of CTBT per disk were: 5 μg (left part in A and B), 10 μg (right part in A, B and C-E).

CTBT has been shown to display cytotoxic activity and the ability to enhance the activity of several antifungal agents at sub-inhibitory concentrations [2]. In disk diffusion assay on YPD medium using the *S. cerevisiae *BY4741 wild type and its *rho*^- ^mutant strains CTBT induced the formation of clear growth inhibition zones that were surrounded by outer inhibition zones corresponding to reduced yeast growth (Figure 1A, B). Growth inhibition zones of respiring cells growing on YPGE medium containing glycerol plus ethanol were significantly larger compared to those with fermenting cells on YPD. This suggested that CTBT disrupted mitochondrial functions (Figure 1C). Essentially the same results were obtained with the BY4742 and FY1678-28C wild type strains (data not shown). On the other hand, *S. cerevisiae *yeast cells grown anaerobically were found to be insensitive to CTBT. As expected for qualitative anaerobicity of our experimental setup, both strains failed to grow anaerobically without ergosterol and unsaturated fatty acids. No inhibition zones were observed when cells of the BY4741 and BY4742 wild type strains were grown in the absence of molecular oxygen on YPD medium supplemented with ergosterol and unsaturated fatty acids (Figure 1D, E). These results clearly indicate that mitochondrial functions and molecular oxygen are involved in the CTBT susceptibility in yeast cells.

### Identification of yeast deletion mutants with increased CTBT susceptibility

To gain more insight into CTBT action we systematically identified mutants with altered sensitivity. We screened the *S. cerevisiae *haploid deletion mutant collection for altered growth in the presence of CTBT. The wild type strain BY4741 from which the EUROSCARF collection has been derived was unable to grow on YPD medium supplemented with 6 μg/ml of CTBT. Therefore, the hypersensitive mutant strains were identified on YPD media containing 2 and 4 μg/ml of CTBT. Using pin replicator, cells of each mutant strain grown in YPD medium containing G418 sulphate were replicated as quadruplets to CTBT containing medium and to YPD control plate. After 6 days of growth the mutant strains sensitive to 2 or 4 μg/ml of CTBT were identified, collected and their sensitivity to CTBT confirmed in independent assays. This screen of the 4700 haploid gene deletion mutants was carried out once and resulted in the isolation of 169 CTBT hypersensitive mutant strains (Additional file 1).

Significantly, some molecular complexes or biochemical pathways were represented by more than one mutant (Table 1). In order to assign cellular functions contributing to CTBT tolerance we searched for enriched gene ontology (GO) categories according to the SGD GO-Termfinder. The most prominent GO terms associated to the genes required for CTBT tolerance were functions related to mitochondria, transcription, DNA repair, and stress response (Table 2).

**Table 1:** Functions of selected genes deleted in CTBT sensitive mutant strains

Function	Gene (ORF) name
Mitochondrial functions	*ADH1, AFG3, ATP1, ATP11, ATP12, ATP18, CIT1, CYT1, DIA4, IMP1, ISA1, ISA2, MAS37, MDM32, MDM38, MGM1, MIP1, MRPL49, MRPS35, MRS1, MSY1, MTG1, OCT1, PCP1, PDC1, PHB1, RML2, RSM19, SOD1, SOD2, TUF1, YDR115W, YGL085W*
Chromatin remodeling and transcription	*ARP5, CDC73, CTK1, HOS2, HPR1, IES6, MED2, MGA2, OPI1, PGD1, RRN10, RME1, ROX3, RSC1, RTF1, RTT109, SGF73, SKN7, SNF2, SPT4, SPT20, SRB5, STP1, SWI3, SWI4, TAF14, THO2, UME6, YAF9, YAP1, YAP7*
DNA repair	*MET18, MMS1, MMS4, MRE11, RAD6, RAD18, RAD50, RAD51, RAD54, RAD57, XRS2*
Lipid metabolism	*AKR1, CHO1, CHO2, DAP1, ERG2, ERG3, ERG6, ERG24, MGA2, OPI1*
Stress response and signal transduction	*ASC1, BCK1, CCS1, CTR1, CYS3, NBP2, REG1, SKN7, SNF1, SOD1, SOD2, YAP1, YAP7*
Vacuolar functions	*AVT4, CWH36, KCS1, TFP1, TFP3, VMA4, VMA21, VMA22*
Protein sorting and degradation	*DIA2, MAP1, PRE9, RAD6, VPS15, VPS20, VPS34*
Amino acid metabolism	*CYS3, ILV1, PRO2, TRP2, TRP3, TRP5*
Transport	AVT4, CTR1, MUP1, TAT1
Mannosyl transferases	*ANP1, OCH1*
Pentose phosphate pathway	*GND1, RPE1*
Ungrouped	*ARD1, BEM1, BIM1, BUB3, BUD25, BUD27, BUR2, CDC50, CIK1, CSM1, CTF18, ENV6, FYV10, GEP4, GET2, HTZ1, KRE28, MTC5, NAT1, NAT3, NCE101, NPT1, NRP1, NUP133, ORM2, PHO85, RAI1, RCY1, REF2, RGI1, RNR4, RPL1B, RPL2A, RPL42B, RTC1, SBH1, YIM2*
Unknown functions	YDR049W, YDR114C, YHR045W, YNR065C, YOR305W

**Table 2:** GO-terms significantly enriched in the 169 genes required for CTBT tolerance (SGD GO-termFinder).

GO_term	Frequency	P-value
**response to stimulus**	27.2%	2.28E-06
**transcription**	23.1%	2.73E-06
**response to stress**	20.1%	6.35E-05
**DNA repair**	10.7%	0.00096
**organelle organization (mitochondrion)**	29.6%	0.0033

The largest group of strains hypersensitive to CTBT contained deletions in genes for mitochondrial biogenesis and functions, including DNA replication (*MIP1*), mRNA processing (*MRS1*), protein synthesis and processing (*AFG3, DIA4, MRPL49, MRPS35, MSY1, MTG1, OCT1, PCP1, RML2, RSM19, TUF1*), respiration (*CYT1*), ATP synthesis (*ATP1, ATP11, ATP12, ATP18*), Fe/S protein biosynthesis (*ISA1, ISA2*), superoxide dismutation (*SOD1, SOD2*) and others.

In the second largest group were mutants in genes involved in gene expression thus hinting at an acute transcriptional response to CTBT. Identified genes are involved in chromatin remodeling (*ARP5, HOS2, HTZ1, RSC1, SGF73, SNF2, SWI3, SWI4, YAF9*), transcription (*CTK1, MED2, ROX3, RRN10, RTF1, SPT4, SPT20, SRB5, TAF14, THO2*) or encode transcription factors involved in oxidative stress response (*YAP1, YAP7, SKN7*) and lipid biosynthesis (*OPI1*).

We identified at least 11 CTBT sensitive strains containing deletion in DNA repair genes, including those involved in homologous recombination and repair (*MMS1, MMS4, RAD50, RAD51, RAD52, RAD57)*, post replication repair (*RAD6, RAD18*), double strand break repair (*MRE11, XRS2*), excision repair (*MET18*) and others. Importantly, deletion of genes encoding functions in lipid metabolism also impaired CTBT tolerance. These are involved in ergosterol (*DAP1, ERG2, ERG3, ERG6, ERG24*), fatty acid (*MGA2*) and phospholipid biosynthesis (*CHO1, CHO2, OPI1*). Along with superoxide dismutase encoding genes, *SOD1 *and *SOD2*, which have a primary role in superoxide radical detoxification, other genes involved in oxidative stress response were also identified. The *CCS1 *gene encodes the specific copper chaperone delivering the copper to Sod1. The *CTR1 *gene is coding for a high affinity copper transporter of the plasma membrane. The transcription factors *YAP1, YAP7 *and *SKN7 *are involved in transcriptional regulation of oxidative stress response genes including *SOD1, SOD2 *and others. Additional functions required for CTBT tolerance involve vacuolar metabolism, protein sorting, amino acid metabolisms and others (Table 1). Importantly, many genes with CTBT defense functions overlap with those involved in menadion, hydrogen peroxide and arsenic stress tolerance [20-23].

The results of phenotypic profiling and the oxygen dependence of CTBT action, led us to the suggestion that CTBT induces reactive oxygen species in yeast cells.

### Transcriptional profile analysis of CTBT treated yeast cells

Profiles of transcriptional responses can be used to identify cellular defense processes. We therefore investigated the immediate response of wild type BY4741 cells treated with CTBT. We used a dose 1/3^rd ^of the minimal inhibitory concentration (6 μg/ml) 2 μg/ml in a time curse of 5, 10, 20 and 40 min in liquid medium. Expression data were collected from duplicate arrays. We identified 314 genes induced in at least one time point by more than 2 fold and 186 genes repressed more than 2 fold (Additional file 2 and 3). Repressed genes comprised many genes with functions in protein biosynthesis (Table 3). This has been observed previously in transcript profiles from cells treated with various other compounds or exposed to stressful conditions [24] and correlates frequently with repression of Sfp1 function [25,26]. Most ribosomal protein genes were repressed to about half of the level of exponentially growing cells.

**Table 3:** GO-terms significantly enriched in the 500 > 2 fold induced or repressed genes (SGD GO-termFinder).

GO_term	Frequency	P-value
**preribosome**	8.2%	1.68E-14
**nucleus**	37.2%	0.00195
**mitochondrion**	61.0%	0.00546
**RNA polymerase complex**	2.0%	0.0093
**response to oxidative stress**	5.40%	2.39E-09
**monosaccharide catabolic process**	3.40%	1.14E-05
**response to chemical stimulus**	11.40%	1.31E-05
**cell redox homeostasis**	1.60%	6.20E-05
**alcohol metabolic process**	6.60%	0.0012

**Figure 2:**
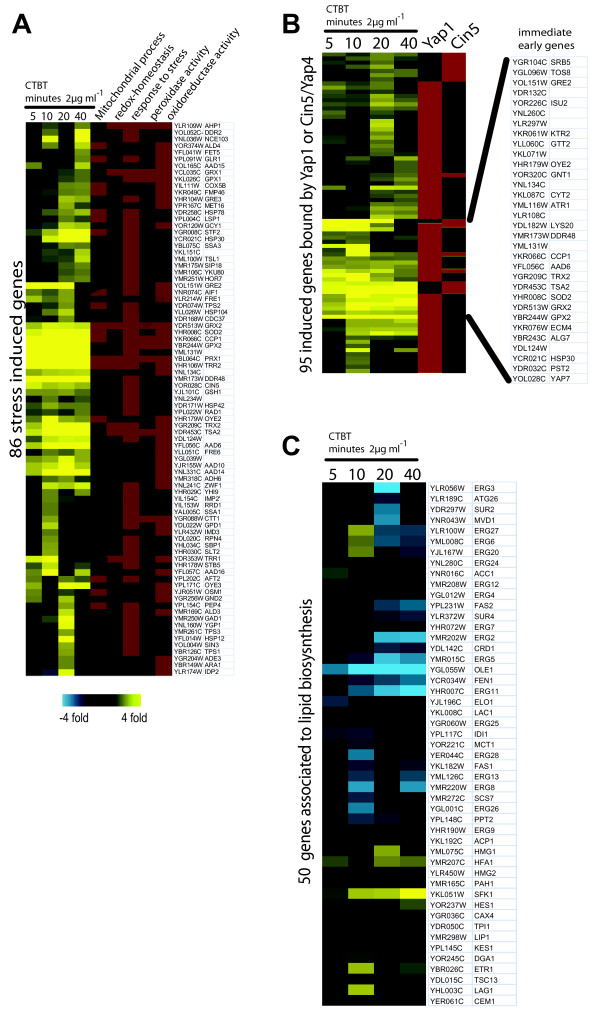
**Transcript profiles of CTBT treated yeast cells**. Exponentially growing cells were treated with 2 μg ml^-1 ^CTBT for the indicated times and microarrays of treated versus untreated cells were done in triplicates. Expression data of genes associated to the GO-term stress response (A), genes regulated and targeted by Yap1 and Cin5 according to Harbison et al., 2004 [27] (B), and the GO-term lipid biosynthetic process (C) were clustered. Enriched GO-terms associated to the respective genes are indicated as colored bars. The full expression dataset is available as supplementary file.

The induction kinetics had an early and a delayed transcriptional wave. Notably, the genes with eminent antioxidant functions were induced most rapidly. Many induced genes were linked to mitochondrial functions and oxidative stress response (Figure 2A, S1). We further predicted the possible transcription factors involved. T-profiler based analysis revealed that among the induced genes some transcription factor binding sites were highly enriched [27,28]. These were the general stress transcription factors Msn2 and Msn4, but more prominently the oxidative stress response factors Yap1, Skn7, Yap7, and Cin5/Yap4 (Table 4). Especially many Yap1 and Skn7 targeted genes were evident in the immediate early response genes. Moreover, a number of Cin5/Yap4 target genes were found to establish a second non-overlapping oxidative stress regulon (Figure 2B). Among the CTBT repressed genes we noticed a number of genes involved in the ergosterol biosynthesis (Figure 2C) and the target of azoles antifungals, the enzyme lanosterol 14-alpha-demethylase Erg11p. To demonstrate a general effect on these genes we show the expression profiles of the genes associated to the GO-term lipid biosynthesis (Figure 2C).

**Table 4:** Transcription factor binding sites enriched in CTBT regulated genes.

Motif	Name	t-value	E-value	Mean	ORFs
**TTASTAA**	YAP1	4.91	1.30E-04	0.713	82
**AGGGG**	MSN2-4	4.72	3.40E-04	0.611	120
**MTTAYRTAAK**	CIN5	4.46	1.20E-03	1.235	17
**CCCCT**	MSN2-4	4	9.20E-03	0.57	118
**CGATGAG**	PAC	-6.48	1.30E-08	-0.344	58
**AAAATTT**	rRPE	-6.57	7.30E-09	-0.039	156

**Figure 3:**
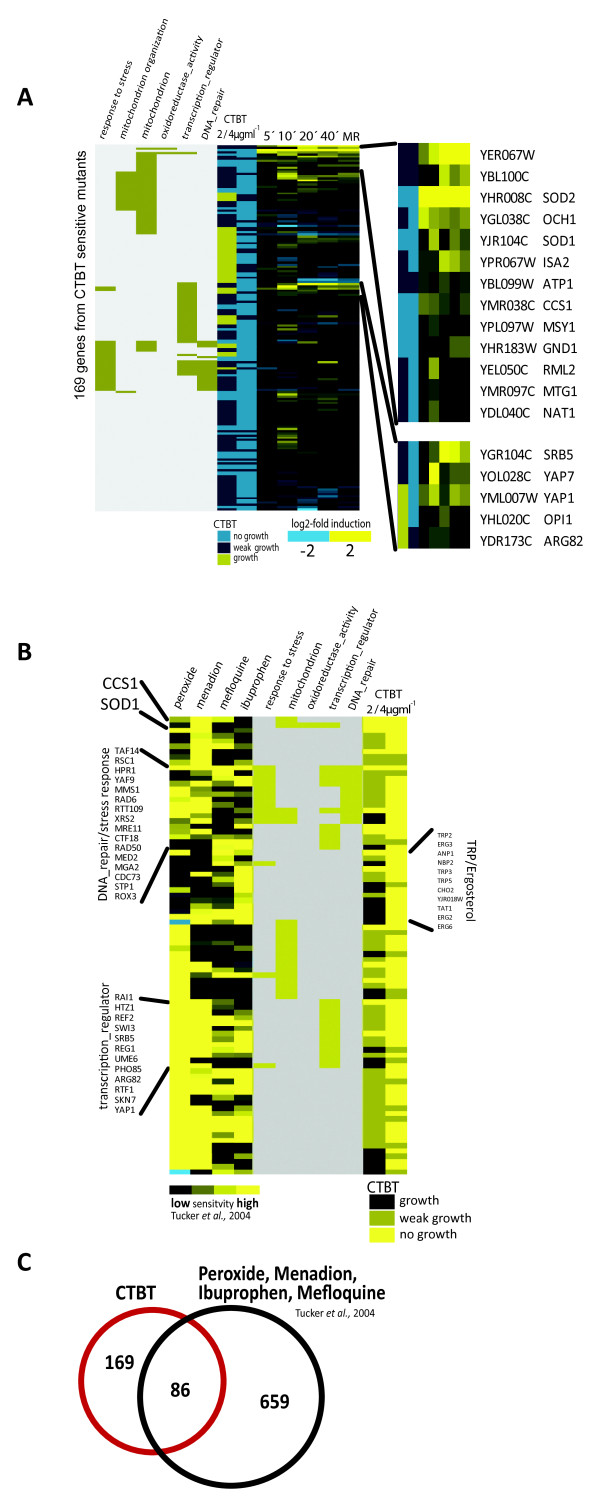
**Comparison of phenotypic and expression profiles**. A) Enriched GO-terms in the 169 genes deleted in the CTBT sensitive mutant strains are indicated in the left panels. Sensitivity levels to 2 and 4 μg/ml CTBT are colour coded as indicated. GO, sensitivity and expression data were hierarchical clustered. Genes belonging to two clusters which are highly expressed and the corresponding mutants highly sensitivity are enlarged and indicate involvement of superoxide dismutases and oxidative stress specific transcription factors. B) Comparison of CTBT sensitive strains to hydrogen peroxide, menadion, mefloquine and ibuprophen sensitive strains. Fitness values (log2 transformed) from [22] were clustered with CTBT sensitivity values (2 very sensitive, 1 sensitive, 0 insensitive). To visualize all genes included in these graphs in TreeView, the raw data are available as supplementary files. C) A Venn diagram shows the overlap between CTBT and datasets from Tucker and Fields [22].

Next we analyzed the connection between transcript and phenotype profiles (Figure 3A). Generally, phenotypic display data do not have large overlaps with transcript profile data. This is due to the fact that pathways activate many target genes in parallel with sometime redundant functions. However, in some cases the overlap is informative since it points to exceptionally important nodes of stress resistance [29]. Here we found a small number of genes both required for tolerance and significantly induced. They encode proteins required for several different mitochondrial functions: *ISA2*, a protein required for maturation of mitochondrial and cytosolic Fe/S proteins, *ATP1*, the alpha subunit of the H^+^ATPase, *SOD1 *the cytosolic superoxide dismutase, *CCS1 *the copper chaperone for Sod1p and *SOD2*, the mitochondrial superoxide dismutase. This overlap points to an essential response to CTBT originating from or localized to mitochondria. Furthermore, the transcription factors Srb5, Yap1 and Yap7 are induced also at the level of transcription (Figure 3A). Comparison of 169 genes required for resistance to CTBT, and 689 required for hydrogen peroxide, menadione, mefloquine, and ibuprophen reistance [22] showed a significant overlap (86 genes, cumulative hypergeometric probability P < 10^-30^; Figure 3B, C). We could not detect a significant bias of genes required for resistance to hydrogen peroxide, menadione, mefloquine, and ibuprophen in our dataset, indicating induction of general oxidative stress by CTBT.

**Figure 4:**
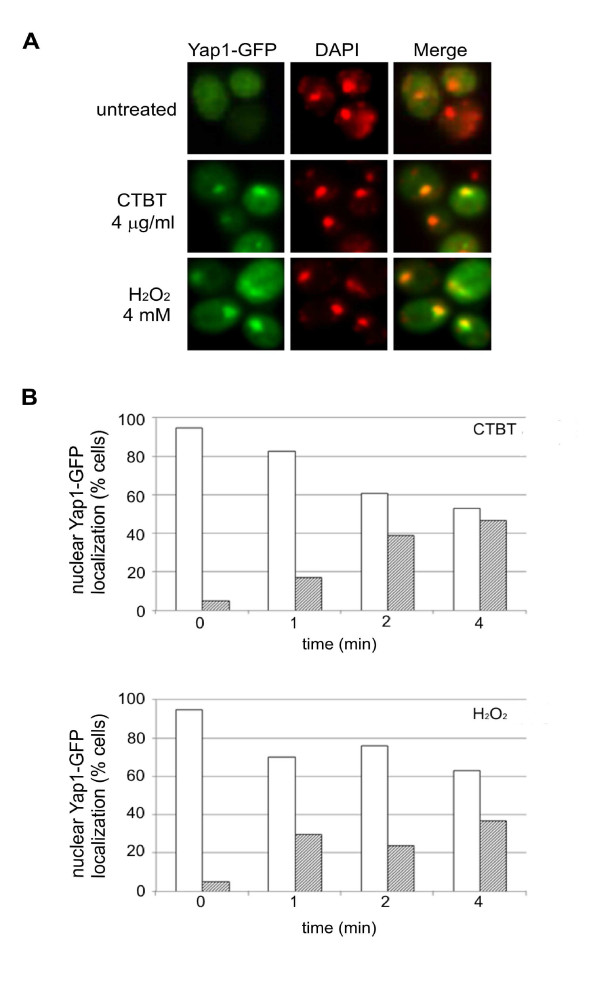
**Analysis of Yap1-GFP localization in CTBT-treated cells**. Exponentially growing yeast cells (EG103) were exposed for indicated time to CTBT (4 μg/ml) and hydrogen peroxide (4 mM) as a positive control. Nuclear localization of Yap1-GFP was verified by co-localization with nuclei stained with DAPI (2 μg/ml) (A). For each sample, at least 100 cells were scored for subcellular localization of Yap1-GFP. Open bars represent cells with cytoplasmic Yap1-GFP, hatched bars represent cells with nuclear Yap1-GFP (B). Data are presented as the average of the two independent experiments.

Yap1 accumulates rapidly in the nucleus in cells exposed to oxidative stress [30]. We followed the intracellular distribution of Yap1-GFP construct and found a similar rapid accumulation of Yap1 in both CTBT and hydrogen peroxide stressed cells (Figure 4A, B). Taken together the transcript profiling data support the phenotypic display by pointing to an immediate oxidative stress response and furthermore to an important protective function of superoxide scavenging and mitochondrial activity.

### Mitochondrial superoxide production and petite mutant formation in CTBT treated yeast cells

**Figure 5:**
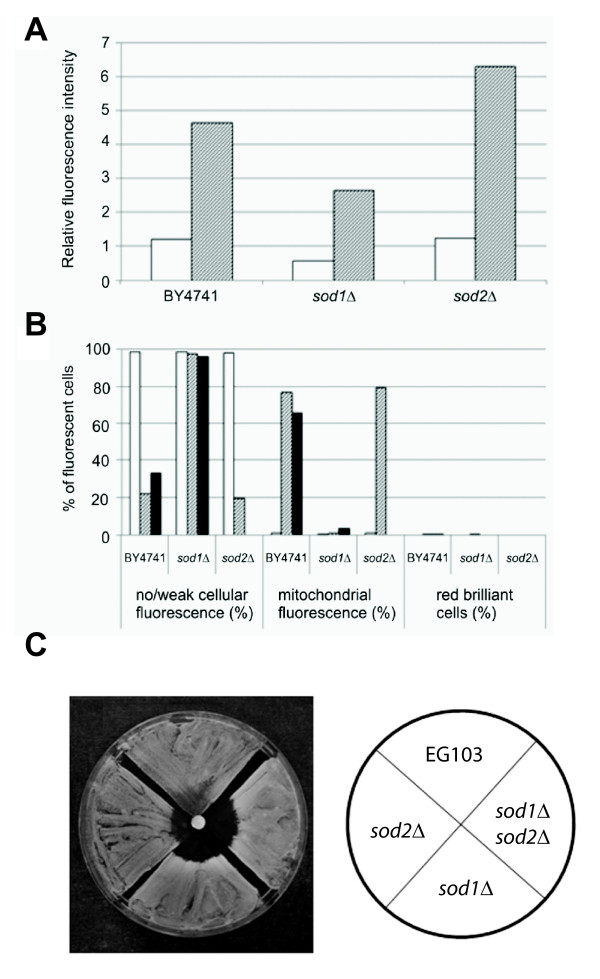
**Superoxide production and growth inhibition in CTBT treated cells**. Fluorescence of oxidized MitoSOX Red was determined using fluorescence spectrometry (A) and fluorescence microscopy (B). The values represent the means of 3 independent experiments. Open bars represent cells grown without CTBT. Wild-type cells (BY4741) were grown in the presence of 2 μg/ml (hatched bars) and 4 μg/ml (filled bars) CTBT. *sod1Δ *mutant cells were grown in the presence of 0.25 μg/ml (hatched bars) and 0.4 μg/ml (filled bars) CTBT. *sod2Δ *mutant cells were grown in the presence of 0.25 μg/ml CTBT (hatched bars). (C) Growth inhibition zones of CTBT in mutant strains lacking the indicated superoxide dismutase genes was scored after 5 days incubation.

*S. cerevisiae *mutant strains deleted in the *SOD1 *or *SOD2 *genes were found to be the most sensitive to CTBT. The minimal inhibitory concentrations of CTBT determined by broth dilution method for the *sod1Δ *and *sod2Δ *mutants (0.5 μg/ml) were 10-times reduced compared to the wild type strain. Thus, we suspected that CTBT induces superoxide formation. To determine this directly, MitoSOX Red was used to assess superoxide radical production in live cells. This dye is selectively targeted to the mitochondria where it is selectively oxidized by superoxide and exhibits red fluorescence upon binding to nucleic acids [31]. As shown in Figure 5A, a large fraction of wild type cells grown for 12 h in YPGal medium in the presence of sub-inhibitory concentrations of CTBT accumulated the dye and fluoresced intensively red, compared with control cells grown in the absence of CTBT. Analysis of individual cells under a fluorescence microscope revealed cells with mitochondrial fluorescence. In addition, a small fraction of cells showed red brilliant fluorescence, which may represent severely damaged or dead cells [32] (Figure 5B). A high percentage of cells exhibiting mitochondrial fluorescence was also observed in the population of the *sod2Δ *mutant strain treated with a 10-times lower concentration of CTBT. In the genetic background of the *S. cerevisiae *EG103 strain the *sod1Δ *mutant cells were significantly more sensitive to CTBT compared with the *sod2Δ *cells. These results demonstrate that CTBT induces an increased production of superoxide that may disturb many cellular functions by damaging nucleic acids, oxidizing proteins and causing lipid peroxidation. In fact, when the *sod2Δ *mutant cells were grown for 24 h in YPD medium containing a sub-inhibitory concentration of CTBT the respiration deficient *petite *mutants were induced in high frequency indicating the damage to mtDNA induced by CTBT (Table 5). Both the CTBT treatment and the absence of Sod1 or Sod2 result in increased ROS formation [33,34]. It was also possible that CTBT mediated inactivation of Sod1p and/or Sod2p might contribute partly to the observed ROS formation. To assess the role of superoxide dismutases, the isogenic series of the *sod1Δ*, *sod2Δ *and *sod1Δ sod2Δ *mutant strains derived from the *S. cerevisiae *EG103 wild type strain were used in zone inhibition assay with CTBT (5 μg per disk) on YPD. We observed that sensitivity of the *sod1Δ sod2Δ *double mutant cells was slightly higher compared to the *sod1Δ *mutant (Figure 5C). We conclude that enhanced ROS production by CTBT treatment is counteracted by both superoxide dismutases.

**Table 5:** Respiration deficient mutant formation in yeast cultures grown for 24 h in YPD medium containing indicated concentrations of CTBT

Strain	CTBT (μg/ml)	*Petite *mutants (%)
BY4741	0	0.2
	2	0.2
	4	0.5
		
*sod1Δ*	0	0.1
	0.25	0.6
		
*sod2Δ*	0	0.1
	0.25	31.7

The results are means of two experiments.

### Theoretical treatment of CTBT activity

In order to get more insight into the mechanism of CTBT induced superoxide generation we used the standard computational protocol to perform quantum chemical calculations of 6 tetrazolo- and triazolobenzotriazines described previously [2]. To compare the important structural parameters and their influence on the biological activity, the unsubstituted [1,2,4] triazolo [3,4, c]benzo[1,2,4]triazine (compound **3**) was added to the original series. Four parameters that could be related to biological activity of compounds were chosen. LogP models the transport of molecules in biological systems, μ is the dipole moment of isolated molecule in Debye units, HOMO (Highest Occupied Orbital) and LUMO (Lowest Unoccupied Orbital) stand for energy of frontier orbitals in eV units.

**Figure 6:**
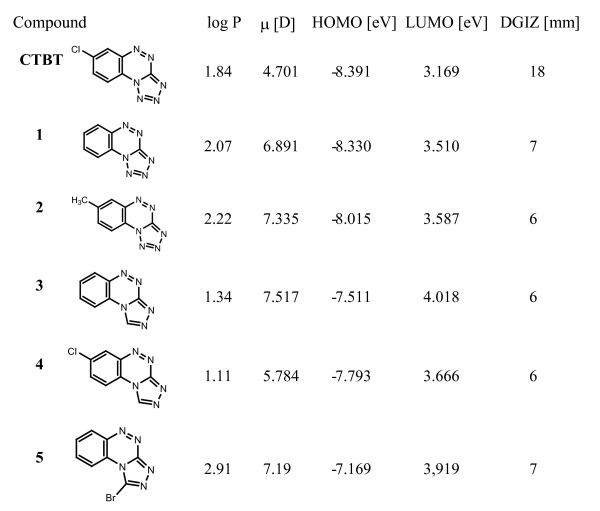
**Structure, calculated theoretical data and growth inhibition caused by CTBT and related compounds**. Log P: partition coefficient in octanol-water system; μ: dipole moment of isolated molecule; HOMO: energy of the Highest Occupied Molecular Orbital; LUMO: energy of the Lowest Unoccupied Molecular Orbital. DGIZ: diameter of growth inhibition in zone inhibition assay on solid medium caused by CTBT [5 μg added to paper disk (diameter of 6 mm)]. **CTBT**: 7-chlorotetrazolo [5,1-c]benzo[1,2,4]triazine; **1**: tetrazolo [5,1-c]benzo[1,2,4]triazine; **2**: 7-methyltetrazolo [5,1-c]benzo[1,2,4]triazine; **3**: [1,2,4]triazolo [3,4, c]benzo[1,2,4]triazine; **4**: 7-chloro[1,2,4]triazolo [3,4, c]benzo[1,2,4]triazine; **5**: 1-bromo[1,2,4]triazolo [3,4, c]benzo[1,2,4]triazine.

As shown in Figure 6, CTBT is predicted to yield the lowest values of dipole moment, HOMO orbital energy and mainly very low value of LUMO orbital energy compared with another compounds under study. The value of LUMO energy appears to be the most important calculated parameter for possible explanation of CTBT activity related to its antifungal effect expressed as the diameter of the growth inhibition zone (DGIZ). If one electron reduction would be assumed as the first step in CTBT mediated superoxide production, the low value of LUMO energy makes the electron transfer into LUMO of CTBT leading to CTBT radical easily possible.

In the chemical structure of studied molecules there are two structural characteristics that decrease significantly the LUMO-energy i.e. chlorine atom and tetrazolo ring that both are incorporated into CTBT. Consequently, CTBT appears as the main candidate for redox cycling and superoxide generation among the studied molecules. These calculations and the genetic data show that CTBT has a capacity to generate superoxide radicals with reducing equivalents possible derived from the respiratory chain.

## Discussion

In this study we show that CTBT, a compound enhancing the antifungal activity of several drugs [2], generates superoxide and other reactive oxygen species (ROS) and induces massive oxidative stress in yeast cells which enhances the antifungal activity of several unrelated drugs.

Five lines of evidence suggest that CTBT produces oxidative stress via generation of superoxide. First, CTBT toxicity required molecular oxygen. Second, it has predicted molecular properties of a molecule capable of redox cycling. Third, we detected oxidative stress using the two *in vivo *reporters MitoSOX Red and Yap1-GFP. Fourth, genetic evidence was provided by the isolation of characteristic mutants with defects in oxidative stress scavenging functions. Fifth, transcription profiling showed activation of regulons associated with oxidative stress response. CTBT activity was strictly dependent on the presence of molecular oxygen because no inhibition of growth by this drug was observed under strictly anaerobic conditions.

Antifungal activity of CTBT was higher on media containing glycerol plus ethanol instead of glucose indicating that developed functional mitochondria might be involved in drug action. This implies that apart from superoxide anion radical (O_2_^.-^) and ROS generation CTBT does not have other direct cytotoxic effects. ROS affect many cellular functions by damaging nucleic acids, oxidizing proteins and causing lipid peroxidation [35,36]. Dismutation of superoxide into hydrogen peroxide and molecular oxygen is catalyzed by two superoxide dismutases: the Cu, Zn-depending Sod1p localized in the cytosol and the mitochondrial intermembrane space and the Mn-depending Sod2p which is localized in the mitochondrial matrix [37]. Among the gene deletion strains selected for increased CTBT sensitivity (Additional file 1), the *sod1Δ *and *sod2Δ *mutant strains were found to be the most sensitive. Additionally, the mutants deleted for *SOD1 *and *CCS1*, a copper chaperone essential for Sod1p maturation, had a similar phenotype. CTBT could act as an inhibitor of Sods. Since the *sod1Δsod2Δ *double mutant cells were also sensitive to CTBT the Sod1p and Sod2p superoxide dismutases cannot be the primary targets of CTBT action. Therefore, CTBT is a producer of superoxide in presence of oxygen.

**Figure 7:**
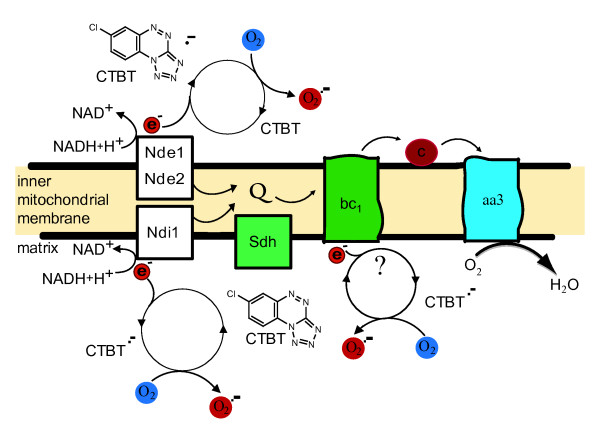
**Scheme of proposed CTBT induced superoxide generation in mitochondria**. Nde, external NADH dehydrogenase; Ndi, internal NADH dehydrogenase; Sdh, succinate dehydrogenase complex; Q, ubiquinone; bc_1_, cytochrome *bc*_1 _complex; c, cytochrome *c*; aa3, cytochrome *c *oxidase. In this model CTBT is reduced to CTBT^.- ^radical by accepting an electron from the NADH dehydrogenases or the cytochrome *bc1 *complex.

CTBT could either directly be a reducing agent or, alternatively, as a cofactor in the context of an enzyme. Our genetic data suggest that CTBT is not acting via a single enzyme because such deletion mutants would be resistant to CTBT and could be easily isolated by genetic means. In fact, in similar screen we were unable to find deletion mutants resistant to CTBT used at the concentration of 10 μg/ml. However, the possibility of a redundant enzymatic activity exists plus the requirement of reducing equivalents. In intact cells, the superoxide anion radical (O_2_^.-^) is the precursor of most ROS and is generated under specific bioenergetic conditions at several sites within the mitochondrial electron-transport system. Most of superoxide is converted to H_2_O_2 _and oxygen inside and outside the mitochondrial matrix by superoxide dismutases [36,38]. Non-mitochondrial sources of ROS involve cytochrome *b*_5 _reductase, NADPH oxidases, lipoxygenases, monoamine oxidases, xanthine oxidase and others [36,37]. In *S. cerevisiae*, the main sites of mitochondrial superoxide formation are one NADH:ubiquinone oxidoreductase located in the inner mitochondrial membrane and facing the matrix [39], two NADH:ubiquinone oxidoreductases facing the mitochondrial intermembrane space and the ubiquinol:cytochrome *c *reductase [40]. This is in line with our scheme proposing CTBT induced superoxide generation in mitochondria (Figure 7). Furthermore, paraquat, the herbicide generating superoxide by redox cycling, is principally reduced by mitochondrial NADH dehydrogenases [41].

Most identified genes required for increased CTBT susceptibility were found to be involved in mitochondrial biogenesis and function, DNA repair, gene expression, lipid metabolism and stress response (Table 1). Many of them are known to be involved in defense processes protecting yeast cells against oxidative stress [35,37] and have been previously identified in genome-wide analyzes of yeast deletion mutant strains sensitive to oxidative stress induced by a superoxide generator menadione, hydrogen peroxide, organic peroxides [20-22], arsenite and cadmium [23].

Remarkable is a high frequency of CTBT hypersensitive deletion mutants with affected mitochondrial functions. A requirement of energy for the repair of oxidatively damaged molecules has previously been proposed to explain why *petite *mutants are more sensitive to oxidative stress than wild type strains [42]. On the other hand one cannot rule out a higher permeability of mitochondrial membranes for CTBT, superoxide or other ROS generated in dysfunctional mitochondria. The increased damage to mutant mitochondria caused by ROS may also reduce the mitochondrial membrane potential under critical level resulting in the arrest of mitochondrial biogenesis required for growth of eukaryotic cells [43].

Other significant pathways involved in CTBT susceptibility were also identified by the presence of several genes involved in the same pathway or encoding the subunits of the particular cellular complexes. This concerns genes involved in the *RAD52 *and *RAD6 *epistasis groups of DNA repair, Paf1 complex of RNA polymerase II (*CDC73, HPR1 *and *RTF1*), protein sorting to vacuole (*VPS15, VPS34*), vacuolar ATPase (*TFP1, TFP3, VMA4, VMA21, VMA22*), N-terminal acetyltransferases (*ARD1, NAT1, NAT3*) acetylating many proteins involved in cell cycle, heat shock resistance, mating, sporulation and telomere silencing as well as genes involved in ergosterol metabolism (*DAP1, ERG2, ERG3, ERG6 *and *ERG24*) and tryptophan biosynthesis (*TRP2, TRP3 *and *TRP5*). Along with tryptophan, interruption of the synthesis of cystein, isoleucin and proline also enhanced the CTBT toxicity. The sensitivity displayed by corresponding mutant strains is apparently not the result of the absence of the amino acids because CTBT was toxic on YPD plates that contain all necessary amino acids. It is possible that the accumulation of intermediates enhances the effect of CTBT.

The overlay of expression data with phenotypic data pointed mainly to superoxide dismutase activity (Sod1, Sod2, Ccs1) and second to the activation of transcription factors Yap1, Cin5/Yap4, and Yap7. Yap1p is a transcription activator involved in the control of multidrug resistance and oxidative stress response [44]. Reactive oxygen species (ROS) generated both from endogenous and exogenous sources induce accumulation of Yap1p in the nucleus resulting in enhanced transcription of many genes involved in removing or detoxifying ROS. Cin5/Yap4 is activated by oxidative stress [45] like Yap1. The contribution of Yap7 to transcription is less understood. Yap7 is involved in transcriptional activation of the *SOD1, SOD2 *and *CCS1 *genes (http://yeastract.com;[27]). However, we found no transcriptional induction of DNA damage specific genes and possibly because this stress type does not surface within the observed time frame. These findings suggest a highly focused primary effect of CTBT on oxidative stress and delayed effects on other pathways.

CTBT enhances activity of several drugs [2]. This synergy becomes perhaps clearer when considering the production of superoxide and other ROS. Our phenotypic screen showed an enhanced sensitivity of mutants in the *RAD52 *and *RAD6 *epistasis groups of DNA repair. 5-Fluorocytosine is a drug which enters nucleotide metabolism and damages the cells by interfering with dNTP and mRNA synthesis. CTBT could act at two levels. Oxidative damage might cause DNA damage and at the same time hamper deoxynucleotide synthesis requiring glutathione or thioredoxin for production via ribonucleotide reductase. Azoles and terbinafin both target ergosterol synthesis. Interestingly, CTBT reduces transcription of most genes for the enzymes of the pathway. Finally, CTBT might exacerbate cycloheximide inhibition on translation by reduction of synthesis of ribosomal protein genes. Oxidative stress causes inactivation of the target of rapamycin complex 1 (TORC1) and thus inactivation of the Sfp1, one major activator of transcription of ribosomal protein genes. An interaction with the popular echinocandines remains to be shown. Up to now the combination of antifungals has been tried *in vitro *in many different combinations. The application of combinations may reduce costs, and importantly shift the effect of the drug towards fungicidal activity (for review see [17]). Apart from combinations of classical antifungals (amphotericin B, azoles, echinocandines), unusual combinations lead to unexpected results as for example in the case of azoles plus calcineurin inhibitors [46] or with membrane active compounds [47].

## Conclusions

CTBT, apart from its weak antifungal activity, is able to strongly inhibit the proliferation of multidrug resistant yeast cells in combination with subinhibitory concentrations of other antifungals. Its mode of action depending on the molecular oxygen has been resolved using the combination of two genome-wide approaches including the screening of yeast deletion library for CTBT hypersensitivity mutants and transcriptome analysis of yeast cells exposed to this drug. We found that CTBT induces an increased production of superoxide and oxidative stress associated with damage to mitochondria and genomic DNA. Yeast cells deleted in nonessential genes encoding proteins involved mainly in mitochondrial function, DNA repair, transcription and oxidative stress response are hypersensitive to CTBT. CTBT rapidly induces transcription of oxidant and stress response defense genes activated mainly by Yap1 and Yap4/Cin5 transcription factors.

The exact molecular mechanism of CTBT action, associated with superoxide generation in mitochondria, is not known so far. It does not require a complete and functional respiratory chain as demonstrated by CTBT sensitivity of *rho*^- ^mutant cells. Theoretical treatment of CTBT activity revealed that this compound might be amenable to one electron reduction. Electrons donated from mitochondrial NADH dehydrogenases or cytochrome *bc*_1 _complex can lead to CTBT anion radical formation that can be re-oxidized by molecular oxygen generating superoxide probable on the both sides of the inner mitochondrial membrane (Figure 7). Our combined genome wide approaches show the power of yeast genetics and transcript profiling to define mode of functioning of bioactive substances.

## Methods

### Strains and culture conditions

The following yeast strains were used:*S. cerevisiae *strains FY1679-28C (*MATa ura3-52 trp1-63 leu2-1 his3-20*) [48], BY4741 (*MATa his3Δ1 leu2Δ0 met15Δ0 ura3Δ0*), BY4742 (*MATa his3Δ1 leu2Δ0 met15Δ0 ura3Δ0*), the complete set of deletion mutants derived from the haploid strain BY4741 (EUROSCARF, http://web.uni-frankfurt.de/fb15/mikro/euroscarf), EG103 (*MATα leu2-3,112 his3Δ1 trp1-289 ura3-52*), EG118 (EC103 with *sod1ΔA::URA3*), EG110 (EC103 with *sod2::TRP1*), EG133 (EC103 with *sod1ΔA::URA3 sod2::TRP1*) [49]. A plasmid expressing an N-terminal GFP-Yap1 fusion was obtained from M. Toledano [50]. Cells were grown in YPD medium containing 2% (w/v) glucose, 1% (w/v) yeast extract, 2% (w/v) peptone, in YPGal medium (as YPD but 2% (w/v) galactose instead of 2% glucose), in YNB medium containing 2% (w/v) glucose 0.67% yeast nitrogen base without amino acids (Difco), in YPGE medium (as YPD but 2% glycerol plus 2% ethanol instead of 2% glucose). The media were solidified with 2% (w/v) bacto agar. Where appropriate, amino acids, uracil, ergosterol (20 μg/ml), Tween 80 (0.06%, w/v) or G418 sulphate (200 μg/ml) was added. For induction of *rho*^-^*/rho*^0 ^mutants, cells were grown in YPD containing of ethidium bromide (25 μg/ml) or CTBT for 24 h, diluted and plated onto solid YPD. Frequency of respiration deficient mutants in yeast culture was determined after staining colonies with TTC (triphenyltetrazolium chloride) or replica plating onto YPGE plates.

### Drug susceptibility testing

Susceptibility of yeast cells to CTBT was determined using the spot test assay. Aliquots of yeast cultures were spotted onto YPD plates containing the indicated concentrations of CTBT. Plates were incubated at 30°C for 6 days. In liquid media, susceptibilities to CTBT were assayed by the broth microdilution method in 96 well plate containing 200 μl YPD supplemented with different concentrations of CTBT. The growth at 30°C was scored after 24 and 48 h. Susceptibility to CTBT was also assessed using zone inhibition assays. Approximately 10^7 ^cells were plated onto YPD media, the filter discs (diameter of 6 mm) soaked with indicated amounts of CTBT were placed on the plates which were incubated at 30°C for 3-6 days before determination of the diameter of the zone of growth inhibition.

### Screening for altered CTBT susceptibility

The collection of viable gene deletion mutants in the BY4741 background was screened for both CTBT hypersensitive and CTBT resistant strains. EUROSCARF mutant strains were transferred from 96 well master plates to solid YPD media supplemented with G418 sulphate. After 3 days, cells from grown colonies were inoculated into the corresponding wells of a 96 well microtiter plates containing 200 μl YPD supplemented with G418 sulphate. Cells were cultured 24 h at 30°C, diluted 20-times in YPD medium containing G418 sulphate and replica pinned onto YPD control plates and plates containing different concentration of CTBT (2 and 4 μg/ml) using a 96 floating pin replicator. The mutant strains were arranged in quadruplet to create a dilution in a given square giving a total of 96 strains plated per agar plate. The plates were incubated at 30°C and scored after 3 and 6 days. The altered sensitivity of strains to CTBT was assessed visually from the growth on the test medium relative to the growth on YPD control plate. To pin-point cellular functions that confer altered CTBT susceptibility, we searched for functional categories in the sensitive gene set according to FunCat at MIPS http://mips.gsf.de. Gene ontology (GO) analysis was done using GO Term Finder in SGD http://yeastgenome.org.

### Microarray analysis

Wild type BY4741 cells were grown for 4 generations in YPD at 30°C to OD_600 _of 1 before CTBT solution (2 mg/ml) was added to a final concentration of 2 μg/ml. After 5, 10, 20 and 40 minutes cells were harvested, washed in ice-cold water and immediately frozen. RNA was isolated by the hot phenol method. 20 μg of total RNA was used for direct labeling cDNA synthesis with either Cy3-dCTP or Cy5-dCTP. Labeled cDNAs were purified with GFX columns (GE Healthcare). Hybridization to cDNA microarrays (Ontario Cancer Institute, Toronto, Canada) was done in triplicates with color inversion in 60 μl DigEasyHyb solution (Roche, Mannheim, Germany) overnight at 37°C. After hybridization, microarrays were washed three times in 1 × SSC, and 0.1% SDS at 50°C for 10 min, followed by 1 min in 1 × SSC und 0.1 × SSC at room temperature and 5 min 500 rpm spin to dryness. Microarrays were analyzed on an Axon 4000B scanner (Invitrogen, Molecular Devices) with Gene Pix Pro 4.1 (Axon; Molecular Probes).

For individual microarrays the intensity of the two fluorescent channels were normalized to the mean of ratio of medians of all unflagged features using the Genepix Pro 4.1 normalization option. Values of not found features were excluded from further analysis. Genes labeled as dubious ORFs in SGD were also removed from analysis. Mean ratios were calculated for features with at least 4 values. The filtered normalized values used for further analysis are available as supplementary file. Cluster analysis [51,52]http://bonsai.ims.u-tokyo.ac.jp/~mdehoon/software/cluster/software.htm was performed using the cluster3 and visualized with TreeView [53]http://jtreeview.sourceforge.net. Significant associations to either GO-terms or transcription factors were obtained by GO-Term Finder at SGD http://www.yeastgenome.org and T-Profiler http://www.t-profiler.org [28]. TreeView files corresponding to the figures are supplied as Additional files 3, 4, 5, 6, 7, 8. Values of genes associated with the most significant terms were visualized by Cluster analysis using complete linkage and correlation as similarity metric. GO assignments were graphically included in the cluster analysis by setting their column weight value to zero. Microarray data have been deposited at ArrayExpress http://www.ebi.ac.uk/microarray with the accession E-MEXP-2307.

### Fluorescence microscopy and spectrometry

Intracellular ROS production was examined using MitoSOX Red (Molecular Probes). MitoSOX Red is a lipid soluble cation that accumulates in the mitochondrial matrix where it can be oxidized to a fluorescent product by superoxide [31]. Yeast strains from initial concentration of 2 × 10^6^cells/ml were grown in YPGal medium containing indicated concentration of CTBT. After 12 h of growth aliquots of 10^9 ^cells were washed twice with phosphate-buffered saline (PBS) and incubated in the dark for 20 min in 5 μM MitoSOX Red. Cells were washed three times with PBS, resuspended in PBS and the percentage of cells positively stained with MitoSOX Red was determined by fluorescence microscopy using a Zeiss Axioplan 2 fluorescence microscope (Thornwood, NY). Images were recorded on fluorescence microscope with a Spot Pursuit camera (Visitron Systems, Puchheim, Germany). Fluorescence of cells was also determined using fluorescence spectrometer (Jasco FP-6300, Tokyo) with excitation and emission wavelengths of 510 and 580 nm, respectively. Nuclei were stained by addition of 1 μl/ml Hoechst 33342 (Molecular Probes). GFP was visualized in live cells approximately 5 minutes after treatment with CTBT without fixation using excitation and emission wavelengths of 355 and 465 nm, respectively.

### Quantum chemical calculations

The usual computational protocol for quantum chemical calculations was used. The optimal geometries of the molecules were obtained by complete geometry optimization employing the AM1 method. This geometry was used as input for the single SCF calculations by the ab Initio method (minimal STO-3G basis set) to obtain the energies and wave functions [54].

## Abbreviations

cDNA: complementary DNA; CTBT: 7-chlorotetrazolo [5,1-c]benzo[1,2,4]triazine; DAPI: 4',6-diamidino-2-phenylindole; GFP: green fluorescent protein; GO: gene ontology; HOMO: highest occupied orbital; LUMO: lowest unoccupied orbital; mtDNA: mitochondrial DNA; NADH(P): nicotinamide adenine dinucleotide (phosphate); ORF: open reading frame; PBS: phosphate buffered saline; ROS: reactive oxygen species; SDS: sodium dodecyl sulfate; SGD: *Saccharomyces *genome database; SSC: saline-sodium citrate buffer.

## Authors' contributions

MB, VB, ZO and IH participated on the screening of the yeast deletion library. MB and JG carried out the fluorescence microscopy and spectrometry. MB prepared RNA and CS performed the transcriptome analysis, data analysis and graphical presentation. PZ carried out the quantum chemical calculations. JS conceived of the study, participated in its design, analyzed data and with CS prepared the last version of the manuscript. All authors read and approved the final manuscript.

## Supplementary Material

Additional file 1 **CTBT sensitive mutant strains**. A pdf file containing all identified CTBT hypersensitive gene deletion mutants. Click here for file

Additional file 2 **Clustered CTBT induced genes**. Contains the graphics of the clustered transcript profile results from 500 significantly induced or repressed genes. Click here for file

Additional file 3 **Cluster data for figure S1**. Contains the text information to reconstruct figure S1 with TreeView. Click here for file

Additional file 4 **Cluster data for figure 2A containing genes associated to the GO-term stress response**. Contains the text information to reconstruct figure 2A with TreeView. Click here for file

Additional file 5 **Cluster data for figure 2B containing genes regulated and targeted by Yap1 and Cin5**. Contains the text information to reconstruct figure 2B with TreeView. Click here for file

Additional file 6 **Cluster data for figure 2C containing genes associated to the GO-term lipid biosynthetic process**. Contains the text information to reconstruct figure 2C with TreeView. Click here for file

Additional file 7 **Cluster data for figure 3A showing enriched GO-terms in 169 genes required for CTBT resistance**. Contains the text information to reconstruct figure 3A with TreeView. Click here for file

Additional file 8 **Cluster data for figure 3B comparing CTBT sensitive strains to hydrogen peroxide, menadion, mefloquine and ibuprophen sensitive strains**. Contains the text information to reconstruct figure 3B with TreeView. Click here for file
