# Supplementary material for: Chemogenomic and transcriptome analysis identifies mode of action of the chemosensitizing agent CTBT (7-chlorotetrazolo[5,1-c]benzo[1,2,4]triazine)
Source: BMC Genomics. 2010 Mar 4;11:153. doi: 10.1186/1471-2164-11-153 (PMC2841119; doi:10.1186/1471-2164-11-153)
Supplement: Additional file 1 — CTBT sensitive mutant strains. A pdf file containing all identified CTBT hypersensitive gene deletion mutants. [file 1471-2164-11-153-S1.PDF]

Table S1. List CTBT sensitive mutant strains. +, growth; sl, slow growth; -, no growth.

ORF	Gene name	CTBT		Description
		2 ug/ml	4 ug/ml	
YOL086C	*ADH1*	-	-	Alcohol dehydrogenase
YER017C	*AFG3*	+	-	Component of the mitochondrial inner membrane m-AAA protease
YDR264C	*AKR1*	sl	-	Palmitoyl transferase involved in protein palmitoylation required for endocytosis
YEL036C	*ANP1*	sl	-	Subunit of the alpha-1,6 mannosyltransferase complex
YHR013C	*ARD1*	sl	-	Subunit of the N-terminal acetyltransferase NatA (Nat1p, Ard1p, Nat5p)
YDR173C	*ARG82*	+	sl	Inositol polyphosphate multikinase (IPMK)
YNL059C	*ARP5*	-	-	Nuclear actin-related protein involved in chromatin remodeling
YMR116C	*ASC1*	sl	-	G-protein beta subunit and guanine nucleotide dissociation inhibitor for Gpa2p
YBL099W	*ATP1*	sl	sl	Alpha subunit of the F1 sector of mitochondrial F1F0 ATP synthase
YNL315C	*ATP11*	-	-	Molecular chaperone, required for the assembly of alpha and beta subunits into the F1 sector of mitochondrial F1F0 ATP synthase
YJL180C	*ATP12*	-	-	Protein required for assembly of alpha and beta subunits into the F1 sector of mitochondrial F1F0 ATP synthase
YML081C-A	*ATP18*	+	sl	Subunit of the mitochondrial F1F0 ATP synthase
YNL101W	*AVT4*	+	sl	Vacuolar transporter, exports large neutral amino acids from the vacuole
YJL095W	*BCK1*	+	-	Mitogen-activated protein (MAP) kinase kinase kinase
YBR200W	*BEM1*	sl	sl	Protein containing SH3-domains, scaffold protein for complexes that include Cdc24p, Ste5p, Ste20p, and Rsr1p
YER016W	*BIM1*	sl	sl	Microtubule-binding protein
YOR026W	*BUB3*	sl	sl	Kinetochore checkpoint WD40 repeat protein
YER014C-A	*BUD25*	sl	-	Protein involved in bud-site selection
YFL023W	*BUD27*	+	sl	Protein involved in bud-site selection, nutrient signaling, and gene expression controlled by TOR kinase
YLR226W	*BUR2*	sl	-	Cyclin for the Sgv1p (Bur1p) protein kinase
YMR038C	*CCS1*	-	-	Copper chaperone for superoxide dismutase Sod1p
YCR094W	*CDC50*	sl	-	Endosomal protein that regulates cell polarity
YLR418C	*CDC73*	sl	-	Constituent of Paf1 complex with RNA polymerase II, Paf1p, Hpr1p, Ctr9, Leo1, Rtf1 and Ccr4p
YER026C	*CHO1*	-	-	Phosphatidylserine synthase, functions in phospholipid biosynthesis
YGR157W	*CHO2*	+	-	Phosphatidylethanolamine methyltransferase (PEMT)
YMR198W	*CIK1*	sl	-	Kinesin-associated protein
YNR001C	*CIT1*	sl	-	Citrate synthase

YCR086W	*CSM1*	sl	-	Nucleolar protein that binds Mam1p at kinetochores during meiosis I to mediate accurate chromosome segregation
YMR078C	*CTF18*	+	-	Subunit of a complex with Ctf8p, required for sister chromatin cohesion
YKL139W	*CTK1*	sl	-	Catalytic (alpha) subunit of C-terminal domain kinase I (CTDK-I) involved in phosphorylation of RNA polymerase II
YPR124W	*CTR1*	-	-	High-affinity copper transporter of the plasma membrane
YCL007C	*CWH36*	sl	-	Dubious ORF, overlapping YCL005W-A encoding subunit e of the V-ATPase V0 subcomplex
YAL012W	*CYS3*	-	-	Cystathionine gamma-lyase
YOR065W	*CYT1*	sl	-	Cytochrome c1, component of the mitochondrial respiratory chain
YPL170W	*DAP1*	-	-	Heme-binding protein involved in regulation of cytochrome P450 protein Erg11p
YOR080W	*DIA2*	+	-	Origin-binding F-box protein that forms an SCF ubiquitin ligase complex
YHR011W	*DIA4*	sl	-	Probable mitochondrial seryl-tRNA synthetase
YEL059W	*ENV6*	sl	sl	Involved at the late endosome and vacuole interface
YMR202W	*ERG2*	+	-	C-8 sterol isomerase
YNL280C	*ERG24*	sl	-	C-14 sterol reductase, acts in ergosterol biosynthesis
YLR056W	*ERG3*	sl	-	C-5 sterol desaturase
YML008C	*ERG6*	+	-	Delta(24)-sterol C-methyltransferase
YIL097W	*FYV10*	+	sl	Protein of unknown function, involved in proteasomal ubiquitin dependent protein catabolic process
YHR100C	*GEP4*	sl	-	Protein of unknown function, required for respiratory growth
YER083C	*GET2*	sl	sl	Subunit of the GET complex
YHR183W	*GND1*	-	-	6-phosphogluconate dehydrogenase (decarboxylating)
YGL194C	*HOS2*	+	-	Histone deacetylase
YDR138W	*HPR1*	sl	-	Subunit of THO/TREX complexes that couple transcription elongation with mitotic recombination and with mRNA metabolism and export
YOL012C	*HTZ1*	sl	-	Histone variant H2AZ
YEL044W	*IES6*	sl	-	Protein that associates with the INO80 chromatin remodeling complex under low-salt conditions
YER086W	*ILV1*	sl	-	Threonine deaminase, catalyzes the first step in isoleucine biosynthesis
YMR150C	*IMP1*	+	sl	Catalytic subunit of the mitochondrial inner membrane peptidase complex
YLL027W	*ISA1*	-	-	Mitochondrial matrix protein involved in biogenesis of the iron-sulfur (Fe/S) cluster of Fe/S proteins
YPR067W	*ISA2*	sl	-	Protein required for maturation of mitochondrial and cytosolic Fe/S proteins
YDR017C	*KCS1*	+	-	Inositol hexakisphosphate (IP6) and inositol

				heptakisphosphate (IP7) kinase
YDR532C	*KRE28*	sl	-	Protein of unknown function
YLR244C	*MAP1*	-	-	Methionine aminopeptidase
YMR060C	*MAS37*	sl	-	Component of the Sorting and Assembly Machinery (SAM or TOB complex) of the mitochondrial outer membrane
YOR147W	*MDM32*	-	-	Mitochondrial inner membrane protein with similarity to Mdm31p
YOL027C	*MDM38*	sl	-	Mitochondrial inner membrane protein
YDL005C	*MED2*	-	-	Subunit of the RNA polymerase II mediator complex
YIL128W	*MET18*	sl	-	DNA repair and TFIIH regulator
YIR033W	*MGA2*	-	-	ER membrane protein involved in regulation of *OLE1* transcription
YOR211C	*MGM1*	sl	-	Mitochondrial GTPase related to dynamin
YOR330C	*MIP1*	sl	-	Catalytic subunit of the mitochondrial DNA polymerase
YPR164W	*MMS1*	sl	-	Protein that acts with Mms22p in a DNA repair pathway
YBR098W	*MMS4*	-	-	Subunit of the structure-specific Mms4p-Mus81p endonuclease
YMR224C	*MRE11*	-	-	Subunit of a complex with Rad50p and Xrs2p (MRX complex)
YJL096W	*MRPL49*	+	sl	Mitochondrial ribosomal protein of the large subunit
YGR165W	*MRPS35*	sl	-	Mitochondrial ribosomal protein of the small subunit
YIR021W	*MRS1*	+	sl	Protein required for the splicing of two mitochondrial group I introns (BI3 in COB and AI5beta in COX1)
YPL097W	*MSY1*	-	-	Mitochondrial tyrosyl-tRNA synthetase
YDR128W	*MTC5*	sl	-	Protein of unknown function, involved in maintenance of telomere capping
YMR097C	*MTG1*	sl	-	Peripheral GTPase of the mitochondrial inner membrane
YGR055W	*MUP1*	-	-	High affinity methionine permease
YDL040C	*NAT1*	sl	-	Subunit of the N-terminal acetyltransferase NatA (Nat1p, Ard1p, Nat5p)
YPR131C	*NAT3*	-	-	Catalytic subunit of the NatB N-terminal acetyltransferase
YDR162C	*NBP2*	+	sl	Protein involved in the HOG (high osmolarity glycerol) pathway
YJL206C-A	*NCE101*	-	-	Protein of unknown function, involved in protein secretion+E121
YOR209C	*NPT1*	-	-	Nicotinate phosphoribosyltransferase
YDL167C	*NRP1*	sl	-	Putative RNA binding protein of unknown function
YKR082W	*NUP133*	sl	sl	Subunit of the Nup84p subcomplex of the nuclear pore complex (NPC)
YGL038C	*OCH1*	sl	-	Mannosyltransferase of the cis-Golgi apparatus
YKL134C	*OCT1*	sl	-	Mitochondrial intermediate peptidase
YHL020C	*OPI1*	+	-	Transcriptional regulator of a variety of genes involved in lipid metabolism

YLR350W	*ORM2*	sl	-	Evolutionarily conserved protein with similarity to Orm1p, involved in response to unfolded proteins
YGR101W	*PCP1*	sl	-	Mitochondrial serine protease
YLR044C	*PDC1*	+	-	Major of three pyruvate decarboxylase isozymes
YGL025C	*PGD1*	sl	-	Subunit of the RNA polymerase II mediator complex
YGR132C	*PHB1*	sl	-	Subunit of the prohibitin complex (Phb1p-Phb2p), inner mitochondrial membrane chaperone
YPL031C	*PHO85*	sl	-	Cyclin-dependent kinase
YGR135W	*PRE9*	sl	-	Alpha 3 subunit of the 20S proteasome, the only nonessential 20S subunit
YOR323C	*PRO2*	+	-	Gamma-glutamyl phosphate reductase
YCR066W	*RAD18*	sl	-	Protein involved in postreplication repair
YNL250W	*RAD50*	+	-	Subunit of MRX complex, with Mre11p and Xrs2p
YER095W	*RAD51*	-	-	Strand exchange protein, forms a helical filament with DNA that searches for homology
YGL163C	*RAD54*	sl	-	DNA-dependent ATPase
YDR004W	*RAD57*	sl	-	Protein that stimulates strand exchange by stabilizing the binding of Rad51p to single-stranded DNA
YGL058W	*RAD6*	sl	-	Ubiquitin-conjugating enzyme (E2)
YGL246C	*RAI1*	sl	-	Nuclear protein that binds to and stabilizes the exoribonuclease Rat1p
YJL204C	*RCY1*	-	-	F-box protein involved in recycling plasma membrane proteins
YDR195W	*REF2*	sl	-	RNA-binding protein involved in the cleavage step of mRNA 3'-end formation prior to polyadenylation
YDR028C	*REG1*	-	-	Regulatory subunit of type 1 protein phosphatase Glc7p
YER067W	*RGI1*	sl	sl	Putative protein of unknown function
YGR044C	*RME1*	+	sl	Zinc finger protein involved in control of meiosis
YEL050C	*RML2*	sl	-	Mitochondrial ribosomal protein of the large subunit
YGR180C	*RNR4*	+	-	Ribonucleotide-diphosphate reductase (RNR), small subunit
YBL093C	*ROX3*	+	-	Subunit of the RNA polymerase II mediator complex
YJL121C	*RPE1*	+	-	D-ribulose-5-phosphate 3-epimerase
YGL135W	*RPL1B*	sl	-	N-terminally acetylated protein component of the large (60S) ribosomal subunit
YFR031C-A	*RPL2A*	+	sl	Protein component of the large (60S) ribosomal subunit
YHR141C	*RPL42B*	-	-	Protein component of the large (60S) ribosomal subunit
YBL025W	*RRN10*	sl	-	Protein involved in promoting high level transcription of rDNA
YGR056W	*RSC1*	sl	sl	Component of the RSC chromatin remodeling complex
YNR037C	*RSM19*	sl	-	Mitochondrial ribosomal protein of the small subunit
YOL138C	*RTC1*	sl	sl	Protein of unknown function
YGL244W	*RTF1*	sl	-	Subunit of the RNA polymerase II-associated Paf1 complex
YLL002W	*RTT109*	sl	-	Histone acetyltransferase

YER087C-A	*SBH1*	+	sl	Beta subunit of the Sec61p ER translocation complex (Sec61p-Sss1p-Sbh1p)
YGL066W	*SGF73*	sl	sl	Subunit of SAGA histone acetyltransferase complex
YHR206W	*SKN7*	+	sl	Nuclear response regulator and transcription factor
YDR477W	*SNF1*	sl	-	AMP-activated serine/threonine protein kinase
YOR290C	*SNF2*	sl	-	Catalytic subunit of the SWI/SNF chromatin remodeling complex
YJR104C	*SOD1*	-	-	Cytosolic superoxide dismutase
YHR008C	*SOD2*	-	-	Mitochondrial superoxide dismutase
YOL148C	*SPT20*	-	-	Subunit of the SAGA transcriptional regulatory complex
YGR063C	*SPT4*	sl	-	Protein involved in the regulating Pol I and Pol II transcription
YGR104C	*SRB5*	sl	-	Subunit of the RNA polymerase II mediator complex
YDR463W	*STP1*	sl	sl	Transcription factor, activates transcription of amino acid permease genes
YJL176C	*SWI3*	sl	-	Subunit of the SWI/SNF chromatin remodeling complex
YER111C	*SWI4*	+	-	DNA binding component of the SBF complex (Swi4p-Swi6p)
YPL129W	*TAF14*	-	-	Subunit of TFIID, TFIIF, INO80, SWI/SNF, and NuA3 complexes
YBR069C	*TAT1*	+	-	Amino acid transport protein for valine, leucine, isoleucine, and tyrosine
YDL185W	*TFP1*	sl	-	Vacuolar ATPase V1 domain subunit A
YPL234C	*TFP3*	sl	-	Vacuolar ATPase V0 domain subunit c'
YNL139C	*THO2*	+	sl	Subunit of the THO complex which is required for efficient transcription elongatuion
YER090W	*TRP2*	-	-	Anthranilate synthase, catalyzes the initial step of tryptophan biosynthesis
YKL211C	*TRP3*	+	-	Bifunctional enzyme exhibiting both indole-3-glycerol-phosphate synthase and anthranilate synthase activities
YGL026C	*TRP5*	sl	-	Tryptophan synthase involved in tryptophan biosynthesis
YOR187W	*TUF1*	sl	sl	Mitochondrial translation elongation factor Tu
YDR207C	*UME6*	sl	-	Key transcriptional regulator of early meiotic genes
YGR105W	*VMA21*	+	sl	Integral membrane protein that is required for vacuolar H+-ATPase (V-ATPase) function
YHR060W	*VMA22*	sl	-	Peripheral membrane protein that is required for vacuolar H+-ATPase (V-ATPase) function
YOR332W	*VMA4*	sl	-	Subunit E of the eight-subunit V1 peripheral membrane domain of the vacuolar H+-ATPase (V-ATPase)
YBR097W	*VPS15*	sl	-	Myristoylated serine/threonine protein kinase
YMR077C	*VPS20*	+	-	Myristoylated subunit of ESCRTIII, the endosomal sorting complex
YLR240W	*VPS34*	sl	-	Phosphatidylinositol 3-kinase
YDR369C	*XRS2*	sl	-	Protein required for DNA repair
YNL107W	*YAF9*	sl	-	Subunit of both the NuA4 histone H4 acetyltransferase complex and the SWR1 complex
YML007W	*YAP1*	+	-	Basic leucine zipper (bZIP) transcription factor

YOL028C	*YAP7*	sl	-	Putative basic leucine zipper (bZIP) transcription factor
YMR151W	*YIM2*	+	-	Dubious open reading frame, overlapping the *IMP1* promoter
YBL100C		sl	sl	Dubious open reading frame, overlapping the *ATP1* gene
YDR008C		sl	-	Dubious open reading frame, overlapping the *TRP1* gene
YDR049W		sl	-	Zinc finger protein
YDR114C		+	sl	Putative protein of unknown function, overlapping YDR115w
YDR115W		+	sl	Putative mitochondrial ribosomal protein of the large subunit
YGL024W		sl	-	Dubious open reading frame, overlapping the *PGD1 gene* involved in RNA polymerase II mediator complex
YGL085W		sl	sl	Putative mitochondrial protein of unknown function, induced in response to MMS
YGR064W		sl	-	Dubious open reading frame, overlapping the *SPT4* gene
YHR045W		-	-	Putative protein of unknown function, synthetically lethal with *ERG11*
YJL120W		+	-	Dubious open reading frame, overlapping the *RPE1* promoter
YJL175W		sl	-	Dubious open reading frame, overlapping the *SWI3* gene
YJL182C		-	-	Dubious open reading frame, overlapping the YJL181w promoter
YJR018W		+	-	Dubious open reading frame, overlapping the *ESS1* gene involved in regulation of the RNA polymerase II phosphorylation
YMR031W-A		sl	-	Dubious open reading frame
YNR065C		sl	sl	Protein of unknown function
YOR305W		+	sl	Protein of unknown function, putative mitochondrial protein
YOR331C		sl	-	Dubious open reading frame, overlapping the *VMA4* gene encoding subunit of the V-ATPase V1 subcomplex
