# Supplementary figures and images for: Chemogenomic and transcriptome analysis identifies mode of action of the chemosensitizing agent CTBT (7-chlorotetrazolo[5,1-c]benzo[1,2,4]triazine)
Source: BMC Genomics. 2010 Mar 4;11:153. doi: 10.1186/1471-2164-11-153 (PMC2841119; doi:10.1186/1471-2164-11-153)

Figure S1

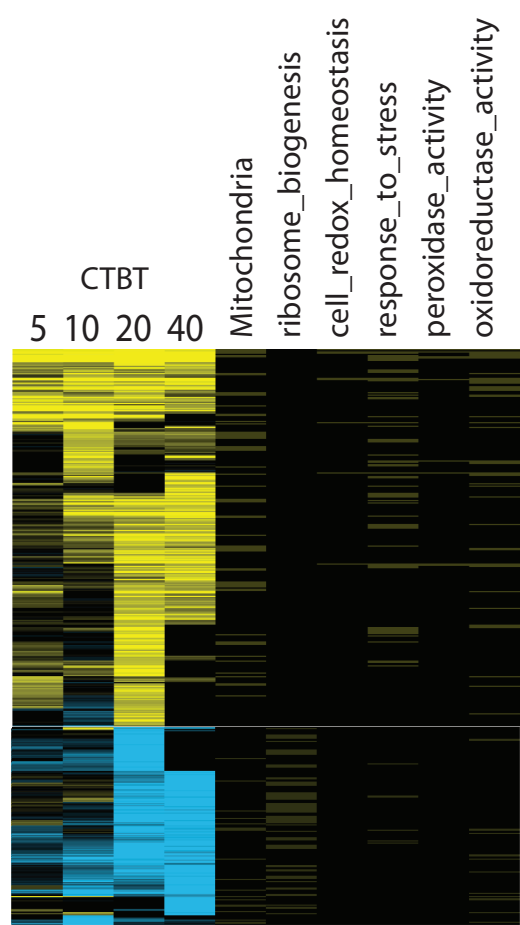

Supplement: Additional file 2 — Clustered CTBT induced genes. Contains the graphics of the clustered transcript profile results from 500 significantly induced or repressed genes. [file 1471-2164-11-153-S2.PDF]
